# The value of a peer-to-peer teaching community in medical education

**DOI:** 10.1186/s12909-026-08642-9

**Published:** 2026-01-31

**Authors:** Alexandra M. Cardoso Pinto, Andrea Perez Navarro, Niamh Aideen Heneghan, Anouk Wijeratne, Ria Varma, Arjun Agarwal, Siddhant Patki, Keir Bhaskar, Joyal Tom, Shila Uruci, Sohag Saleh, Ana Baptista

**Affiliations:** Imperial College School of Medicine, Imperial College London, London, UK

**Keywords:** Peer-assisted learning, Near-peer learning, Medical education, Undergraduate medical education, Clinical education, Medical students, Communities of practice, Mixed methods research

## Abstract

**Introduction:**

The Medical Education Society (MedED) at Imperial College School of Medicine (ICSM) offers near-peer educational opportunities across all years of medical school. Near-peer education has demonstrated significant benefits in medical education. However, studies have yet to explore the value of establishing a peer-to-peer teaching community.

**Methods:**

Medical students who participated in MedED as student-attendees or student-teachers during the academic year 2022-23 were invited to participate in a survey and follow-up interview, exploring their experiences within the Society. Survey data was collected anonymously through Qualtrics, and interviews were held on Microsoft Teams. Quantitative survey data was analysed using descriptive statistics, while interview transcripts and free-text survey responses underwent inductive thematic analysis.

**Results:**

A total of 66 students completed the survey, with 19 (28.8%) from years 1–2 and 47 (71.2%) from years 3–6. Early-year students had higher lecture attendance rates (79%) compared to later-year students (34%), and both groups preferred online rather than in-person lectures (both > 50%). For student-attendees, benefits of participating in MedED included improving knowledge, motivation and sense of community. Among student-teachers, main motivations for teaching included helping others and developing transferrable skills.

A total of 13 participants were interviewed, including 5 who were both student-attendees and student-teachers. Three themes emerged: academic value, highlighting knowledge and skills gained through MedED; career prospects, focusing on long-term professional benefits; and sense of community and wellbeing, emphasising the positive social interactions and support networks fostered through MedED.

**Conclusion:**

MedED provides student-led teaching initiatives that supplement the formal curriculum, enhancing student confidence and inclusivity, and fostering a sustainable community of peer-education. Beyond immediate academic values, this community has also created longer-term, personal and professional impacts on students, including broadening career aspirations. This work highlights opportunities for further development through student-staff collaborations and the role of peer communities in supporting student wellbeing.

**Supplementary Information:**

The online version contains supplementary material available at 10.1186/s12909-026-08642-9.

## Introduction

Peer-to-peer and near-peer education have shown significant benefits in medical education. Real-world studies have highlighted that such educational approaches enhance both the teaching and learning experiences. For the students who teach, it improves their own understanding of the material, helps build confidence and provides opportunities to apply and enhance their skills as educators. For the learners, it provides a more relatable and less intimidating learning environment, often leading to better academic outcomes. According to social learning theory [1], the ability of students to more closely identify with their peers increases their credibility as role models, hence strengthening their ability to successfully pass information on. By creating a more conductive environment for positive behaviour modelling, peer-to-peer education initiatives help enhance student’s learning experience [2]. 

Considering these benefits, multiple student-led educational initiatives have emerged in medical schools across the world [3–7]. One of such initiative is the Medical Education Society (MedED) running at Imperial College School of Medicine (ICSM), in London (United Kingdom) since 2014. All medical students at ICSM automatically become members of MedED on starting their course, and through them gain access to a wide range of educational opportunities, including lectures, small-group tutorials, clinical skills teaching, mock exams, and exam question banks amongst other resources - all of which are created, led and disseminated by undergraduate medical students. The focus of these opportunities is not only to help students better understand medical and surgical concepts included in their university curriculum but also to provide advice with regards to exam preparation, workload management, and performance in clinical placements. Medical students at ICSM have the opportunity to engage with the Society all throughout their medical school experience, which allows the Society to create a greater sense of community amongst its members based on the reciprocity and collaboration within peer-to-peer education.

Beyond direct benefits of having a space to learn relevant curriculum material and advice delivered by peers, there are benefits in the establishment of this peer learning community. According to social learning theory [8], observing and interacting with near-peers is an important part of development of healthcare professionals and educators. For students who attend teaching, this may include observing near-peers as role models in their approach to learning, clinical placements or even extra-curricular activities such as medical education; for students who teach, this may involve belonging to a space that enables them to build confidence in their knowledge and skills, and develop professionally through interaction with a greater range of colleagues. Whilst the academic benefits of peer-to-peer and near-peer education in medical education have been explored more widely [9, 10], no formal study has been conducted yet to understand the possible wider value of creating this community of peer teaching within ICSM or elsewhere.

## Aims & objectives

The aim of this study is to understand the value of creating a community of peer-to-peer and near-peer teaching (and learning) amongst medical students. This includes the following objectives:

- Describe the impact, both academic and wellbeing-related, of attending near-peer teaching programmes delivered by MedED at ICSM.
- Contextualise these findings through a framework of value creation [11].
- Identify ways in which students could benefit more, as attendees and as teachers, from MedED at Imperial College London School of Medicine.

### Methods

A mixed-methods approach, including a survey and follow-up semi-structured interview, was followed.

### Data collection and recruitment

Medical students from all year groups [1–6] who attended any events organised by the Medical Education Society (MedED) as either attendees or teachers at ICSM during the academic year 2022-23, were invited to take part.

Students were asked to consider the completion of an online, anonymous survey (Supplement 1) hosted on Qualtrics at the end of each lecture series or mentorship scheme delivered by MedED during the 2022/2023 academic year. Students were recruited to participate through a variety of methods, including formal invitation at the end of a lecture series or talk, social media communications and emails to those who subscribed to MedED mailing-list. The recruitment message included an information sheet and details on who to direct questions to.

Those who completed the survey were redirected to an optional sign-up form for an interview through another anonymous Qualtrics form. Sign-up details were unpaired to survey results to ensure anonymity of responses. Those who responded were emailed a date for the interview, along with another information sheet and consent form to be reviewed and signed prior to the interview.

Interviews were semi-structured (Supplement 2) and conducted on Microsoft Teams, using Imperial College London accounts. Interviews were audio and visually recorded, with written consent from participants. Interview participants were offered a £10 Amazon voucher as a token of appreciation for their time.

Survey and interview questions have not been previously published and can be found in Supplements 1 and 2, respectively.

### Data storage and protection

All data for this study was stored on secure Imperial College London servers. Interview recordings were transcribed anonymously and then deleted. Survey results were stored on Imperial College Qualtrics.

### Data analysis

Quantitative survey data was analysed using descriptive statistics on Microsoft Excel. Interviews were transcribed verbatim and anonymised, after which they were coded on NVivo 14 following an inductive approach to thematic analysis, with Braun and Clarke’s stages of thematic analysis [12] used as guidance. Two initial transcripts were coded collaboratively by two researchers, following which one researcher coded the interview and the second reviewed these codes. Themes were then reviewed by other co-authors. Thematic analysis was also performed to responses to the free-text questions of the survey.

### Ethics approval

This study was reviewed and approved by the Imperial College London Education Ethics Review Process (EERP2223-032).

## Results

### Survey results

A total of 66 students completed the survey. Of these, 19/66 (28.8%) were student attendees in their early years, prior to any large clinical placements (years 1 or 2), and 47/66 (71.2%) were in their later years, where most of their learning happens through clinical placements (years 3, 5 or 6). Details of the Imperial College MBBS curriculum are publicly available [13] online. Thirty-one respondents (31/66, 47.0%) also answered in quality of student-teachers.

Respondent demographic characteristics are summarised in Table 1.

**Table 1:** Survey respondent demographics

	Student attendees		Student teachers *N* (%)
	Early years (pre-clinical) *N* (%)	Later years (clinical) *N* (%)	
N	19	47	31
Year of study			
First	13 (68.4)	-	0 (0)
Second	6 (31.6)	-	2 (6.5)
Third	-	16 (34.0)	7 (22.6)
Fourth (iBSc)	-	4 (8.5)	4 (12.9)
Fifth	-	16 (34.0)	9 (29.0)
Sixth	-	11 (23.4)	9 (29.0)
Gender			
Female	2 (10.5)	11 (23.4)	10 (32.3)
Male	16 (84.2)	35 (74.5)	19 (61.3)
Non-binary	1 (5.3)	1 (2.1)	2 (6.5)

#### Student attendees

Lecture attendance was significantly higher amongst early-year students (79% of early-year students reported attendance rates of > 75% compared to 34% of later-year students). Both early and later-year students preferred online lectures.

**Fig. 1:**
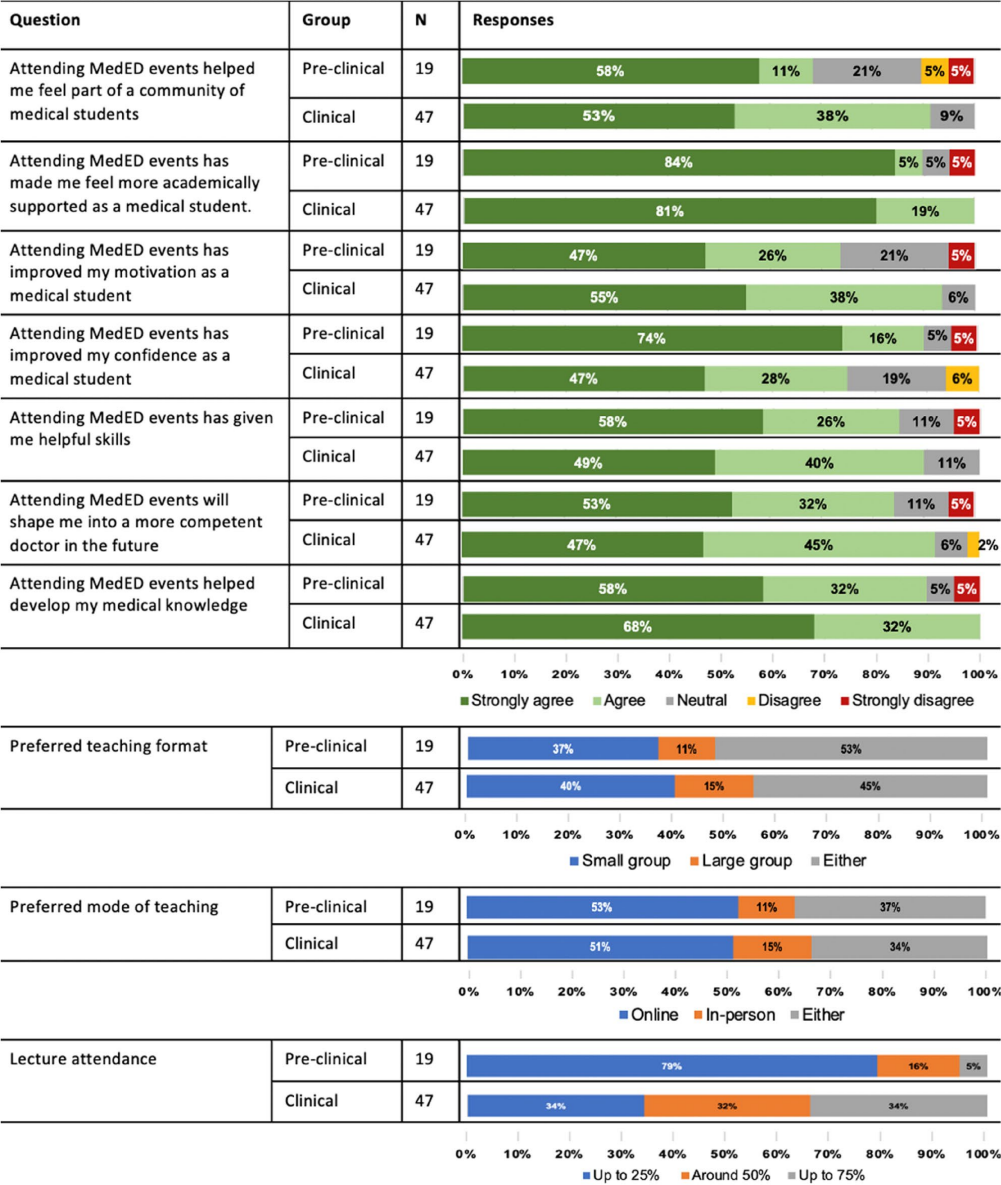
Survey responses for student attendees

Most students strongly agreed with statements that attending MedEd lectures improved their medical knowledge and skills, confidence as a medical student and future doctor, and motivation, as well as helping them feel more academically supported and part of a community (Fig. 1). The prospect of receiving good quality academic support was the most common motivator for lecture attendance amongst both pre-clinical (10/19, 52.6%) and clinical students (28/47, 59.6%). Knowledge improvement (12/19 pre-clinical, 63.2% vs. 39/47 clinical students, 83.0%) and increased confidence (4/19 pre-clinical, 21.1% vs. 11/47 clinical students, 23.4%) were the most cited perceived benefits of lecture attendance. Both student groups identified the introduction of additional physical learning resources – particularly exam questions – and new learning initiatives as the main potential improvement for the Society. Responses are summarised in Table 2.

**Table 2:** Student-attendee open-ended survey responses

Theme	Early years (Pre-clinical) *N* (%)	Later years (Clinical) *N* (%)
Benefits		
Confidence	4 (21.1)	11 (23.4)
Exam technique	1 (5.3)	4 (8.5)
Knowledge	12 (63.2)	39 (83.0)
Motivation	2 (10.5)	2 (4.3)
Revision* strategies	2 (10.5)	9 (19.1)
Welfare	2 (10.5)	5 (10.6)
Community	0	3 (6.4)
Efficiency	0	1 (2.1)
Interactivity	0	1 (2.1)
Learning resources	0	4 (8.5)
Reputation	0	1 (2.1)
Student perspective	0	1 (2.1)
Motivators		
Academic support	10 (52.6)	28 (59.6)
Concise/targeted summaries	3 (15.8)	6 (12.8)
Practice questions	2 (10.5)	0
Accessibility	1 (5.3)	0
Contextualisation	0	1 (2.1)
Reputation	0	14 (29.8)
Revision strategy	0	3 (6.4)
Teaching skills	0	1 (2.1)
Improvements		
Accessibility	1 (5.3)	0
Physical resources	1 (5.3)	4 (8.5)
Breaks	1 (5.3)	0
Content	3 (15.8)	2 (4.3)
Practice questions	3 (15.8)	2 (4.3)
Welfare	1 (5.3)	5 (10.6)
Anonymity	0	1 (2.1)
In-person events	0	1 (2.1)
Recordings and slides	0	3 (6.4)
Schedule	0	2 (4.3)
Learning events	0	5 (10.6)

* “Revision” in the context of this work refers to the process of going back over previously learned content to strengthen understanding, improve recall and/or correct misunderstandings. This process can take a variety of learning strategies

#### Student-teachers

**Fig. 2:**
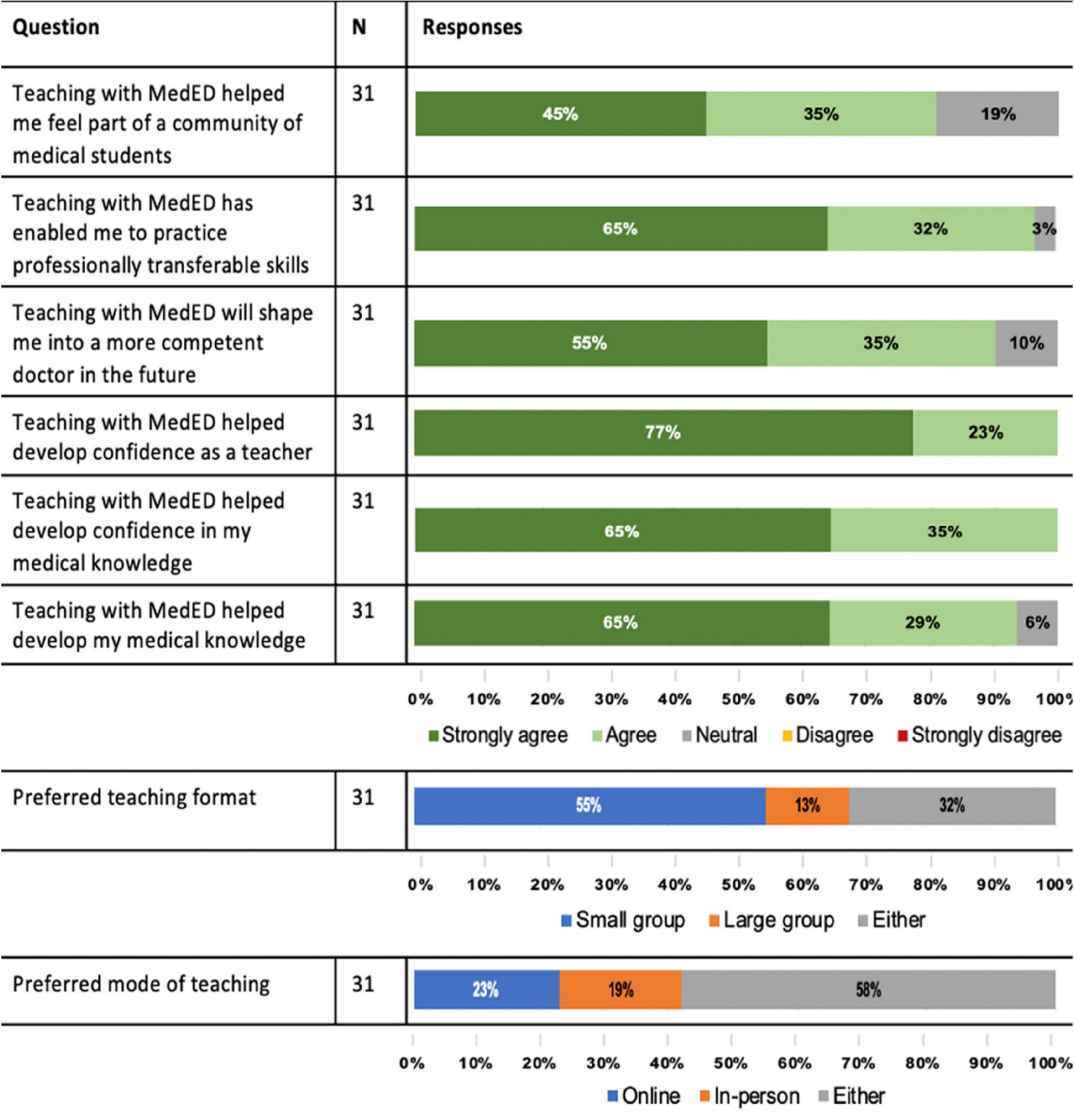
Summary of survey responses by student-teachers

All students (31/31, 100%) completing the survey who reported previous teaching within the Society, also completed the survey as teachers. Most students agreed strongly with the short and long-term benefits of teaching with MedED (Fig. 2).

The most common motivator for student-teachers to engage with MedED’s teaching activities was the desire to help others (16/31, 51.6%) followed by the prospect of developing transferrable skills (10/31, 32.3%). The most commonly listed of such skills was the ability to create successful teaching resources (13/31, 41.9%), followed by a range of soft professional skills such as organisation and time management (12/31, 38.7%), teaching skills (9/31, 29%), presentation and public speaking skills (7/31, 22.6%), communication skills (6/31, 19.4%), leadership (4/31, 12.9%), and teamwork (3/31, 9.7%). The main challenges associated with being a student-teacher included time (11/31, 36.7%) and workload (9/31, 30%) management (Table 3).

**Table 3:** Student teachers open-ended survey responses

Themes	Teachers *N* (%)
Motivation	
Being part of a community	2 (6.5)
Consolidating knowledge	1 (3.2)
Enjoyment of teaching	9 (29.0)
Future career	3 (9.7)
Helping others	16 (51.6)
Skill attainment	11 (35.5)
• *Communication skills*	*6 (19.4)*
• *Confidence*	*5 (16.1)*
• *Critical thinking*	*1 (3.2)*
• *Knowledge*	*4 (12.9)*
• *Leadership*	*4 (12.9)*
• *Organisation*	*6 (19.4)*
• *Patience*	*1 (3.2)*
• *Presentation*	*7 (22.6)*
• *Research*	*1 (3.2)*
• *Resourcefulness*	*1 (3.2)*
• *Self-motivation*	*1 (3.2)*
• *Teaching resource design*	*13 (41.9)*
• *Teaching skills*	*9 (29.0)*
• *Teamwork*	*3 (9.7)*
• *Time management*	*8 (25.8)*
Improvements	
Confidence	1 (3.2)
Content management	4 (12.9)
Expectations	2 (6.5)
Workload	9 (29.0)
Interactivity & engagement	6 (19.9)
Logistics	2 (6.5)
Scheduling	6 (19.4)
Time management	11 (35.5)
Welfare	2 (6.5)

### Interview results

A total of 13 participants were interviewed, including five (38.4%) who provided the perspective of both student attendees and student-teachers, the remainder being student-attendees only.

A total of four (30.8%) were male and nine (69.2%) were female. Four (30.8%) were in their second year, two (15.4%) in their fourth year, five (38.4%) in their fifth year, and two (15.4%) in their final year. In other words, six (46.2%) were only able to provide early-years, pre-clinical perspective, and seven (53.8%) were able to also focus on the value of MedED during clinical years.

**Fig. 3:**
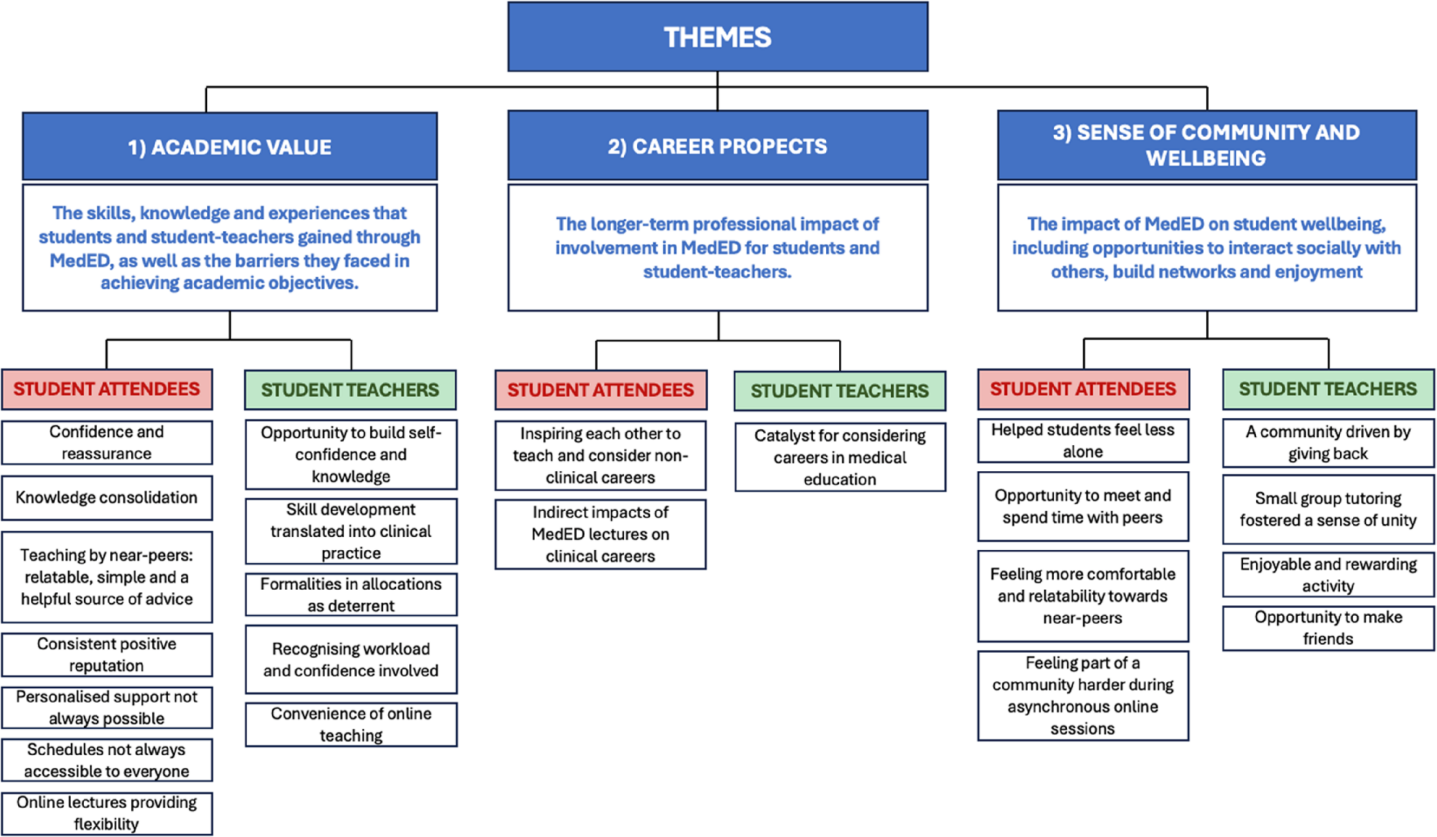
Summary of interview themes

Three key themes were identified across interviews with students and student-teachers (Fig. 3).

Each of these themes is explored below from the perspective students and student-teachers, respectively.

## Students

### Academic value

Students highlighted MedED as source of confidence, knowledge consolidation, and helpful resources. This was compounded by its positive reputation amongst students. However, some also acknowledged that teaching sessions were not always personalised to student needs, and may not be accessible to all. Whilst online lectures enabled more consistent access for some, a subset of participants continued to prefer in-person sessions. All of these findings are explored below.

#### MedED teaching offered confidence and reassurance

Participants reported repeatedly attending MedED events as these provided them with confidence and reassurance. For example, “[MedED lectures] made me feel a lot more reassured in terms of knowing what the year is like, and whatever the task or exam entailed.” (Participant 3).

Without this reassurance, participants reported “stressing about [not knowing] what I need to know” (Participant 3) and instead feeling “more confidence on the subjects” (Participant 1). Part of this confidence gain was possible as MedED provided opportunities to receive “direct feedback” and “having a feel of what the actual exam will be like” through provision of mock exams and small-group sessions (Participant 4).

Another part of this reassurance and confidence, came from near-peers providing advice on what is “really high-yield knowledge and comes up a lot in the exam”, without which students would feel “alone” and overwhelmed as there is “just so much content” and uncertain over “what do I need to know?” (Participant 5). This finding highlighted that there was an educational gap that MedED came to fill. As Participant 8 highlighted, “Sometimes I feel like the [formal MBBS] lecturers don’t really explain it very well. Or they rush through the content. And just go into the [MedED] tutorials and having them being like condensed and have their content be in like a very concise format really helped me with my revision.”

Similarly, the nature of the MBBS programme is that different students will likely receive different teaching during clinical placements as there is “[not] much centralised teaching on a lot of the topics” (Participant 4), particularly during clinical years. However, this brings a sense of uncertainty and concern that students may miss an important part of the curriculum and therefore “attending the [MedED] lectures gives you the sense of covering the most important concepts of the different specialties” (Participant 4).

#### Near-pear teaching as a method of consolidating knowledge

Most MedED lectures and tutorials cover content that has already been covered in the formal medical school curriculum. It is not meant to act as a replacement but instead it is an “opportunity to go through the same topic, which you’ve already seen, but with a group of students” (Participant 2) and therefore “good for consolidating concepts” (Participant 10). As Participant 4 described, “hearing things multiple times can sort of reinforce your learning and sometimes having things described in different ways might reinforce your learning as well”.

In addition to lectures and tutorials, MedED provides additional learning resources also created by near-peers, such as guides, mock exams and videos. One student describes one of the guides as being “very helpful to me; I used it, pretty much, as one of my main resources” (Participant 2).

Lectures also incorporate exam-style questions, which provides “lots of opportunities to test yourself in an interactive way” (Participant 2), an effective form of active learning. On the other hand, some students found the more passive nature of near-peer lectures as an opportunity to “just sit there and still take on board a lot of the stuff they say without necessarily having that feeling like you’re sitting down to revise” (Participant 5). This may be especially helpful for those who feel unable to engage in active learning at the end of a work day, but still wishing to revise.

#### Teaching by near-peers: relatable, simple and a helpful source of advice

Teaching in MedED is led by near-peers, that is, older year students sharing their knowledge and experience with younger years. This was often described as very beneficial, given their relatability. For example, “they know what it’s like to struggle with the content. They know what it’s like to, you know, be a student at that time. They know, like, what problems they had” (Participant 6).

Additionally, “the lecturers are often quite good at breaking [learning content] down and seeing it in a way that makes me understand it.” (Participant 5). Their ability to “condensing what you need to know about conditions” (Participant 3) was frequently highlighted.

Furthermore, attending teaching by near-peers can be a helpful way of developing new revision strategies, for example through “learning new ways of memorising things, learning student techniques, perhaps some acronyms that students might use” (Participant 2).

#### Recognising meded’s consistent positive reputation

Participants reported knowing about MedED’s positive reputation prior to attending any lectures; “I had heard from my friends that they were really good which is why I went” (Participant 10). Others described MedED as a “kind of a panmedical school society that everyone knows about, a lot of people are part of” (Participant 9), seen as a “provider of high-quality teaching” (Participant 7), giving a sense of security that it was worth their attendance. When this was confirmed following their attendance, participants felt like they could “come back and know [to] expect good teaching as well” (Participant 7).

Several students spoke highly of MedED during the interview, with one participant especially highlighting the impact of this group on generations of students: “Like the amount of people that it’s impacting, and the amount of help that it’s giving (…) I think it’s a really invaluable resource, and it should be continued for future generations (…)” (Participant 1). Nevertheless, despite the positive impacts, there were also several challenges highlighted during the interviews, outlined below.

#### MedED May not always be able to provide personalised learning support

Whilst lectures can be helpful to consolidate knowledge throughout medical school, the same cannot be said for the intercalated BSc where students undertake individual written assignments. In that situation, MedED “can’t really help because, it’s your own data and they can’t like sit down with you and go through it” (Participant 3).

Similarly, attending lectures may not always be the most effective way of learning for some students, especially those who already feel confident in the subject of the session. Instead, for example “practice questions (…) [may be a] more valuable” form of revision (Participant 6), as it may be more efficient and promote active learning.

#### Lecture schedules not always accessible to everyone

Most MedED lectures happen on weekday evenings. Those who travel far from clinical placements or university lectures may end up missing the lecture. Similarly, many students have extra-curricular commitments or personal plans in these hours. As a result, students often rely on lecture recordings: “I think I’ve definitely watched more recordings than attend the live sessions, but more because (…) even if I wanted to attend the live session, sometimes I had other things going on” (Participant 8).

Furthermore, it is also important to consider that students might feel “exhausted by the end of the day” and hence prefer to watch the recordings at another time (Participant 8). Others might simply feel like after a full day of placement they would prefer “to do stuff that wasn’t related to medicine because (…) I needed that sort of break and it was like it was way too intense for me to attend a lecture (…)” (Participant 6).

#### Online lectures providing flexibility to meded’s students

Whilst traditionally MedED lecture were held in-person, since the COVID-19 pandemic these have entirely shifted online. This remained post-lockdown as it provided greater convenience to students. For instance, for those with alternative plans for their evening, it meant they did not have to “change my plans to go to a MedED event” (Participant 5), as they can access slides and recordings at a later point.

However, recordings lose some of the interactivity and engagement that a live session may provide, “especially with Mentimeter to go alongside a session” as a platform to anonymously ask attendees questions (Participant 8). This interactivity also means students “concentrate better” (Participant 8). However, if stressed about exams, the recordings feel “more efficient (…) because I can just play it in 2x speed” (Participant 8).

For those who live further away, or would like to go home earlier, online lectures also provide that flexibility and ability to attend lectures “from the comfort of your home” (Participant 2). Some also reported feeling “more safe to ask questions [online] than when we’re in a huge lecture hall” (Participant 1). This was not shared by all participants however, with another reporting feeling more comfort participating in small, in-person sessions as “you’re more comfortable to speak up and answer questions, you can raise your hand to give an answer, compared to online” (Participant 2).

### Career prospects

Regularly attending MedED events helped some students begin to reflect on their future careers. This primarily included careers teaching or medical education, but also, to a lesser extent, preparation for their clinical career, as detailed below.

#### Students inspiring each other to teach and consider non-clinical careers

Witnessing near-peers give lectures and tutorials encouraged younger students to do the same. Watching peers “invest[ing] the time and energy to teach me this (…) definitely inspired me to do the same (…) And I think if I wouldn’t have attended those lectures series, I wouldn’t even have thought about being involved in MedED (…)” (Participant 6).

Whilst most students described teaching as something to do whilst in medical school, others – particularly those in older years – began thinking of medical education, or otherwise non-clinical careers, as a possibility for their longer-term career. For example, near-peers also “showed me that there is more to medicine than just doing clinical medicine.” (Participant 10).

#### Acknowledging the indirect impacts of MedED lectures on clinical careers

There were several students who did not necessarily think that MedED events would have direct impacts on their careers: “As I’m still quite early on in my years, I don’t think these first three years have had, you know, massive impact on my career prospects” (Participant 8). Nevertheless, the knowledge gained through MedED may have an indirect impact on their career; “indirectly, it’ll help me in the future because I’ll know more content, and I’ll be able to help my patients better” (Participant 8).

### Sense of community and wellbeing

MedED provided students a chance to meet and engage with near-peers as well as peers from their own year group. This was what enabled a community to form – and there were several wellbeing benefits of this described.

#### Attending MedED lectures helped students feel less alone

Being in a space where “you’ve got lots of people in with you, (…) learning all together” made students feel like they’re “not alone – which is always motivating and helpful” (Participant 2). They also felt like they were “part of the medical student community when attending student-led lectures, MedED lectures, compared to faculty ones” (Participant 2). These group lectures enabled students to realise that they were “going through the same things as people that have done the tasks (…) Like if there’s someone who’s gone through it that can guide you, then that’s helpful” (Participant 3). It was often “nice to feel like other people have been through what you’re going through before (…) I remember after the first PoM [1st year science module] lecture literally crying and being like ‘what is happening, am I just too stupid to be here’ and then you go to a MedED lecture. Everyone’s like ‘nah, everyone is really confused after that lecture. It’s fine’. So it is nice to just feel like there are other people who are like in it with you, you know, and who survived” (Participant 5).

#### Lectures as an opportunity to Meet and spend time with peers

Especially true for in-person lectures, these gave students “another opportunity to speak to peers (…) even in the online ones, it’s nice seeing your friends’ names pop up” (Participant 4). Furthermore, they sometimes presented opportunities to “know more people who had very similar interests to me” (Participant 6). This was especially relevant for students who began the course during COVID-19 and had limited opportunities for engaging with others.

The interaction with peers also means that “you learn a lot quicker (…) because it’s a lot more interactive (…) and you’re answering questions alongside others” (Participant 2).

Similarly, this was an interesting opportunity for networking. For instance, “I managed to meet other people (…) who are interested in MedED (…) it could be potential to like build more projects (…)” (Participant 6). This was particularly relating to buddy schemes and teaching skill training events.

#### Feeling more comfortable and relatability towards near-peers

Some students also reported that MedED lectures created a more comfortable space for asking questions: “I feel like the environment was a bit more open in order to ask questions, not feel like pressured by having someone really like (…) senior, you know, kind of like maybe judging you about the questions” (Participant 6). Furthermore, students described that it was “nice to hear it from students who understood what we had to understand and knew what needed to be explained” (Participant 10).

#### Feeling part of a community is harder during asynchronous and online sessions

One of the participants described not feeling like they were part of a MedED community, and explained that, “I think it’s more me because I didn’t really attend very many live sessions. But I do think like if I do attend start attending more live sessions. It will definitely feel a bit more like I’m studying together with other people (…)” (Participant 8). Similarly, small group tutorials were described as better encompassing this sense of community as it was easier to interact with each other.

Furthermore, this community spirit was easier to feel in-person because “you were seeing friends at those events. I think the online tutorials less so” (Participant 4). Nevertheless, having “older years or [those] who have now graduated taking the time to teach younger students does sort of add to that community feel as well” (Participant 4).

## Student-teachers

### Academic value

Several academic benefits and motivators were also described for student-teachers. These included building self-confidence, knowledge and translational skills. However, these benefits were to be balanced with formalities in allocations and workload involved. There were also important distinctions between online and in-person teaching. All of these findings are explored below.

#### Teaching as an opportunity to build self-confidence and knowledge

The vast amounts of preparation involved in giving a lecture meant that student-teachers found themselves developing deeper levels of understanding of the topics they taught: “I think (…) you have to have a good understanding of something in order to be able to teach it. So it really, it also kind of forces you to make sure that you understand the topic really well” (Participant 4). Similarly, “it helps build your own confidence, seeing that you actually understand those things” (Participant 4).

#### Being a student-teacher enabled the development of various skills which may be translated into clinical practice

In addition to learning and confidence, students reported improving on skills such as “communication skills in general” which also benefit their day-to-day interactions “with patients as well” as they are better able to “explain things in a simple way [which] is useful translated to the wards” (Participant 10). Communicating and working with others, as well as “being able to give feedback to each other” were also important skills to develop (Participant 7).

Furthermore, “delivering a session for large crows of medical students (…) for me it was an opportunity to like step-up the game”, and an opportunity to practice feeling comfortable with public speaking (Participant 7).

Delivering these sessions, and the associated feedback, also “helped me to know more about where my strengths and weaknesses lay (…) and help me to develop better reflection skills. And I think that’s very easily applicable to other parts of medicine” (Participant 7).

Nevertheless, student-teacher participants also described several barriers that challenged the academic value they were able to take from lectures.

#### Formalities in allocations can be a deterrent to teaching with MedED

Whilst student-teachers are given the chance to rank the topics they would prefer to teach, as mapped to the official medical school curriculum, there is no guarantee that everyone will receive their preferred options. As a result, “you might not end up teaching (…) something that you would really enjoy teaching about” (Participant 4). This lack of autonomy over teaching subjects can be frustrating for student-teachers and also limit the quality of the lecture.

Furthermore, all student-teachers are interviewed prior to being selected. Whilst this is to ensure that those who are selected are capable of providing reliable teaching (especially as applications are competitive), it does present an additional barrier. As one participant described, “if I have to go through the effort of preparing for an interview to do that, then I think I’d just rather find other opportunities to teach, even if I don’t get a certificate for it” (Participant 4).

#### Recognising the workload and confidence involved in being a student-teacher

Preparing a teaching session, especially a large-group lecture, requires a lot of effort, which students may not always be able to provide alongside their studies: “I think sometime just time pressures like putting together a lecture can be time consuming, especially during busy parts of the year” (Participant 10). Additionally, even small-group sessions can be logistically challenging to set up, as different students have different availabilities and finding a time where everyone is available “could be a bit of a nightmare” (Participant 4).

Furthermore, several student-teachers feel underconfident in their knowledge, adding to the pressure and hesitancy in taking part in further teaching opportunities. As one described, “probably not actually feeling that I knew enough (…) I never felt confident to sign up to [teaching a lecture]” (Participant 9).

#### Convenience of online teaching

The preparation involved in delivering an in-person or online session is similar, “and you are going to prepare regardless of whether it’s in-person or online” (Participant 4). However, online sessions are “easier to schedule, easier to find the time”, whilst “in-person formats you might do a longer session because it feels like you’ve already gone through the effort of getting to a place, everyone has gone through the effort of getting to some place. So you want to cover a bit more of the content.” (Participant 4).

### Career prospects

A major motivator for joining MedED as a student-teacher was the career prospect. For some, the experience led to further interest in medical education as a career; for others, it offered recognition that would support future work applications.

#### Teaching in MedED as a catalyst for considering careers in medical education

Participants described MedED as having “definitely made me more interested in pursuing something MedEd alongside medicine and it’s made me sort of understand the value of teaching for my own development as well as for other people’s” (Participant 10). One participant described this interest in medical education as something that “I probably wouldn’t have had if I’d only attended faculty lectures alone” (Participant 2) and further explained that “I want to be a teacher as well as a doctor now” (Participant 2).

Teaching with MedED was also recognised as something helpful to add to a CV: “I have to say, part of it was a bit of like, I did want to get some sort of like pride in it (…) I felt like it was going to be quite good for my curriculum (…)” (Participant 6). Another participant described how “it’s quite wide spanning the opportunities that come from teaching” (Participant 7).

### Sense of community and wellbeing

Student-teachers described smaller, in-person group settings as enabling them to feel part of a community; this included communities between student-teachers, and between student-teachers and their students.

#### Small group tutoring fostered a sense of unity

To support students in preparing for practical clinical exams (OSCEs and PACES), MedED arranges small-groups with 1–2 tutors and up to 6 tutees. These sessions provided greater opportunities for students to interact: “It was nice because our tutoring was in a group with friends. So it made me see those friends more as well, and it also felt, I guess very supportive (…)” (Participant 4).

Some student-teachers also “had a little group chat with all the tutors who were going to deliver a session and a lot of times, even if it wasn’t a main group chat, I knew who I could reach out to that were delivering other sessions [and ask for help or feedback]. So, getting a lot of peer feedback within the tutors really created that sense of community (…)” (Participant 7).

Others, however, highlighted that they “haven’t experienced that much collaboration between tutor groups and I think it could be beneficial to have more of that collaboration. Even in terms of creating resources like you know someone might have written really good cases and if you exchange them, you have more content to deliver to your tutees without necessarily putting in that much time into it (…)” (Participant 4).

#### A community driven by giving back

Several participants reported having been inspired to become MedED teachers by watching near-peers. They saw it as a way to “give back (…) to the society and also to other medical students” (Participant 6). MedED not only helped them succeed in exams, but also helped some students find “contacts for the future” and “a lot of people (…) who have acted as mentors (…) I don’t know if that would have been possible without MedED” (Participant 6).

This spirit of giving back established a cycle amongst students. As one participant described, “It feels like you’re helping the community like other students helped you before (…)” (Participant 4). Furthermore, “it’s not about the certificate in the end, it’s (…) about doing something you enjoy and helping others and learning along the way.”

#### Teaching with MedED as an enjoyable and rewarding activity

In addition to the academic and career benefits, being a student-teacher for MedED was also described as a pleasant and rewarding activity: “I found I really enjoyed teaching (…) it felt like a really rewarding thing to do” (Participant 10).

Part of the reward came from watching students succeed. For example, “I think seeing the way the person who’s being taught (…) able to answer [a question] (…) or ask a really good question that you probably hadn’t even thought of before. Or even just seeing your full crowd of people like actively nodding and showing that they understand… For me, that’s what’s most rewarding” (Participant 7). Feedback can also be very motivating, for example “hearing back from people to say, ‘you know what that was really useful and I appreciate your time’. Just the [gratitude for these] experiences is what I find really, really amazing” (Participant 7).

#### Teaching with MedED as an opportunity to make friends

One student-teacher described how “it was really nice getting to know [my tutees]. I didn’t know them before. I now regularly see them at events. I’m always very, very happy to see them” (Participant 4). Someone else described a similar experience; “I actually managed to build like a lot of bonds with [my tutees] and even with my friends as well because it was a different way of like, you know, hanging out with them” (Participant 6). However, this sort of interaction or bonding was harder to achieve online, as “with the in-person sessions you have more of an opportunity to actually socialise, whereas the online ones tend to be very much more focused on the actual cases, the work” (Participant 4).

### Towards a value creation framework: the case of MedED

Following the thematic analysis guided by Braun and Clarke’s framework [12], the results were further interpreted following a value creation framework for communities and networks (Table 4) [11]. 

**Table 4:** Applying a framework of value creation

Framework Value*	Definition**		Identified Value (from interviews)	Example quote
Immediate Value	Immediate, tangible benefits from participating in or being part of MedED.	Students	Confidence and reassurance	“[MedED lectures] made me feel a lot more reassured in terms of knowing what the year is like, and whatever the task or exam entailed.” (Participant 3)
			High yield learning	“Attending the [MedED] lectures gives you the sense of covering the most important concepts of the different specialties.” (Participant 4)
			Interactive and relatable learning space	“They know what it’s like to struggle with the content. They know what it’s like to, you know, be a student at that time. They know, like, what problems they had” (Participant 6)
			Sense of peer-to-peer support	“It is nice to just feel like there are other people who are like in it with you (…).” (Participant 5)
		Student-Teachers	Consolidating knowledge	“(…) You have to have a good understanding of something in order to be able to teach it. So it really, it also kind of forces you to make sure that you understand the topic really well”. (Participant 4)
			Enjoyment while teaching	“I found I really enjoyed teaching (…) it felt like a really rewarding thing to do.” (Participant 10)
			Sense of unity in small-group teaching	“I actually managed to build like a lot of bonds with [my tutees] and even with my friends as well because it was a different way of like, you know, hanging out with them.” (Participant 6)
			Opportunity to receive feedback	“Got my feedback (…) it was really good to hear from the students (…) what they thought [about the teaching session].” (Participant 7)
Potential Value	Perceived future benefits of participating in or being part of MeDED, including resources or skills that can be applied beyond the immediate setting.	Students	Motivation to teach	“I think that attending MedED, just watching older students perform lectures has really motivated me to do the same, so I’m really keen to get involved in delivering lectures, get involved in MedED.” (Participant 2)
			Resources for future	“The resources that MedED produces, for example the CPA guide was very helpful to me. I used it, pretty much as one of my main [revision] resources, alongside the faculty resources.” (Participant 2) “You guys did an OSCE guide. I still use that, even for year 6.” (Participant 9)
			Networking	“I managed to meet other people as well and I think in the future I’ll be able to meet other people who are interested in MedED well, so again it could be potential to build more projects (…) I think it would definitely like open the doors for that (…).” (Participant 6)
		Student-Teachers	Development of transferable skills, including: communication, public speaking, organisation	“I built like a lot of skills. Like I built a lot of like teaching skills, I believe.” (Participant 6) “Teaching helped me with my communication skills in general.” (Participant 10)
			Confidence in knowledge	“And I think also it helps build your own confidence, seeing that you actually understand those things and seeing, for example, of examining, examining in mock CPA’s or OSCE’s.” (Participant 4)
			Understanding one’s strengths and weaknesses	“It’s helped me to know more about where my strengths and weaknesses lay from a self-reflection perspective and also from getting feedback from others.” (Participant 7)
Applied Value	The practical application of what was gained from participating or being part of MedED.	Students	Trying new revision strategies	“I can’t deny it’s definitely helped me with my revision strategies. It’s shown me how asking questions, it can really help you get things stuck in the head. So, I’ve incorporated that into my revision routine (…) it’s given me a new way to revise.” (Participant 1)
			Applying knowledge in clinical placements	“It helped me, I think for being in placement, especially [for] the infinite quizzing from all the registrars and consultants (…).” (Participant 7)
			Applying to become student-teachers	“I’m hoping to deliver some lectures in the future, just wanting to get more involved with Medical Education (…).” (Participant 2)
		Student-Teachers	Showcasing skills and experience in future applications, including further teaching	“Being able to show that I’ve got experience in teaching and especially in the medical setting (…) it’s highly favoured in further career processes and application processes as well. (…) I can (…) apply that to other roles, other leadership opportunities.” (Participant 7)
			Utilising transferable skills in clinical placements	“Knowing how to be able to explain things in a simple way [which] is useful translated to the wards.” (Participant 10).
Realised Value	Outcomes of the applied value.	Students	Preparedness for exams	“CPA like mock sort of like exam. That was yeah, that was really helpful because again I was quite lost. So like I actually understand and I understood like what the stations were about and I was like much less panicked actually.” (Participant 6)
			Becoming a student-teacher	“(…) Just seeing them teaching so well (…) inspired me to do the same for other students and actually get more involved. And I think if I wouldn’t have attended those lecture series, I wouldn’t have even thought about being involved in MedED.” (Participant 6)
		Student-Teachers	Further job opportunities	“It’s quite wide-spanning the opportunities that come from teaching.” (Participant 7)
			Being able to support team members in clinical work	“It’s sort of taught you how to teach and which I think will be very beneficial for in future career because obviously medicine is a, you know, medicine teamwork is really, really important. And teaching is part of that teamwork. And supporting maybe new members of the team or junior members of the team.” (Participant 4)
Reframing Value	Long-term outcomes or changes, such as perspectives, strategies or identities.	Students	Considering broader career paths, including non-clinical careers	“They showed me that there is more to medicine than just doing clinical medicine. I don’t think I’m good for medical education but it’s showed me there’s other bits to medicine outside of just clinical work.” (Participant 10)
			Sense of belonging at medical school	“Being part of that student community, which is always great as a medical student – you feel part of the medical school community when you’re attending student led lectures, MedED lectures, compared to faculty ones.” (Participant 2) “(…) feeling a bit more like you’re not alone in this.” (Participant 6)
		Student-Teachers	Envisaging oneself as a medical education in the long-term	“I want to be a teacher as well as a doctor now.” (Participant 2)

*“Promoting and assessing value creation in communities and networks: a conceptual framework” by Etienne Wenger, Beverly Trayner and Maarten de Laat

**Summary definition, as interpreted by authors and applied to topic of study

The findings derived from thematic analysis of interview transcripts previously described enable the exploration of what might be gained through the collaboration, teamwork and networking within MedED. This may include short- and long-term outcomes, tangible and non-tangible, for both students and student-teachers (Table 4). Whilst some values may be a direct consequence of the content offered by MedED, some may fall beyond this and highlight the benefits that derive through interactions and networking between or amongst students and student-teachers. The next section will explore further learning points from the reconceptualization of the results into this value creation framework.

## Discussion

Overall, the findings from this study indicate that MedED plays a significant role in providing reassurance and confidence to medical students, particularly through near-peer teaching. Students appreciated the additional support in understanding what to expect from their courses and exams, with mock exams and small-group sessions being particularly beneficial. Near-peer teaching was highlighted as an effective method for consolidating knowledge, providing relatable and simplified explanations, and offering valuable revision strategies. MedED’s positive reputation further motivated students to attend, contributing to a sense of community and wellbeing. However, challenges such as the limitations in supporting students requiring more personalised approaches for instance with independent assignments, the accessibility of lecture schedules, and the impact of online versus in-person sessions were noted. For student-teachers, MedED provided opportunities to build confidence, deepen knowledge, and develop various skills, although the preparation involved and formalities in teaching allocations posed some barriers. The experience also inspired some students to consider careers in medical education, fostering a community driven by a desire to give back.

Similar initiatives are taking place in universities internationally. For example, a near-peer anatomy teaching course at the University of Birmingham [14]and another near-peer teaching program at Harvard University [15] where student tutors were paired with faculty members, both showed high success rates in improving confidence and understanding of learning material amongst student attendees and improvement in teaching skills amongst student tutors. A randomised control study at Imperial College London assessing the value of near-peer teaching in a surgical skills course, showed that teaching groups led by a near-peer tutor were associated with higher rates of performance improvement amongst attendees than those led by faculty members [16]. 

When applying the value creation framework by Wenger et al. [11] to the results, it is apparent that several immediate and potential values are identified within both students and student-teachers. This ranges from academic values such as building knowledge and confidence, but also some wellbeing values, including sense of peer-to-peer support and enjoyment of teaching and learning.

MedED offers a unique student-led teaching opportunity that benefits both attendees and student-teachers. It is important to recognise that MedED is not intended to replace the formal curriculum but rather to supplement it. This distinction ensures that students view MedED as an additional resource to enhance their learning, rather than a substitute for their formal education. However, there are some gaps in the formal curriculum that MedED can help address, such as the space for peer-led small-group tutorials to provide practice opportunities for practical exams, the organisation of mock exams and even the creation and provision of mock written exams and question banks for these. MedED has also been described as providing a more comfortable environment for students to ask questions as well as improve the accessibility of lectures, which is particularly important for neurodiverse students who may struggle with the length of lectures or the design of slides [17]. Arguably, however, those are gaps that should not only be covered by a student-led Society, but rather shared with faculty as feedback for urgent improvement.

It is also important to consider the wellbeing role that MedED should hold, which thus far has not been a priority for the Society. The vision of MedED is for it to be a source of support rather than adding pressure for more studying. The demanding nature of medical school already requires a significant amount of independent study, often leading to long hours and a poor work-life balance. It is essential to ensure that MedED does not exacerbate this issue. While the Society’s good reputation and positive word-of-mouth can motivate students to attend, it can also create pressure and a sense of guilt if they do not participate. It is important to counter this by emphasising the voluntary nature of MedED and promoting a healthy balance between study and personal time. This may be a difficult space to navigate: student-teachers prefer higher attendance, but in reality, providing lecture recordings and take-home resources may make sessions more accessible and promote better work-life balance for students. To counter this, it may be helpful for MedED to consider new methods of ensuring students provide feedback for lectures even if they watch a recording, and consider supplementing this with other forms of formal feedback, for instance, supervisor or peer-observer reports [18]. 

There were also some values identified in higher domains of the value creation framework, including some realised and reframing values. These mainly revolved around career prospects for students and student-teachers. More specifically, considering broader career opportunities in medicine beyond the clinical, such as medical education. In this way, MedED also becomes a space for mentorship and development beyond the immediate, academic support.

To make MedED even more valuable, fostering student-faculty partnerships could be highly beneficial [19, 20]. The current system places a significant burden on students and relies heavily on their dedication. By involving staff more actively, the programme can offer professional development opportunities for students, share resources, and ensure better control over the quality and content of the material presented. Additionally, professional development for student-teachers can help them develop teaching skills and confidence, enriching their educational experience and future careers [18, 21]. 

Encouraging student-student collaborations for sharing resources and learning may also add significant value to MedED. By creating a more collaborative environment, students can benefit from diverse insights and study techniques, making learning more inclusive. Moreover, increasing the flexibility of session timings and locations can make MedED more accessible to all students, accommodating different schedules and learning preferences. This flexibility can help ensure that more students can take advantage of the supportive and enriching opportunities MedED provides.

There are limitations to this study that should be acknowledged. Firstly, participation in this study was voluntary, leading to possibility of response bias as those with particularly positive (or negative) experiences may have been more motivated to engage. The overall sample size was relatively small, which may limit the breadth of the perspectives included – which this study hoped to balance through its mixed-methods approach, namely interviews. Finally, although steps were undertaken to ensure rigour in qualitative analysis, the involvement of researchers who were themselves part of the MedED community introduces the possibility of researcher bias influencing data interpretation. Nevertheless, the oversight of two supervisors not related to MedED were helpful in mitigating this.

## Conclusion

To conclude, the student-led Medical Education Society (MedED) provides a valuable student-led teaching opportunity that enhances the educational experience for both attendees and student-teachers. By supplementing the formal curriculum, it addresses gaps such as the opportunity for frequent small-group practice session for practical assessments and mock exams, which are crucial to improving student confidence, and aims to foster an inclusive learning environment by delivering content through methods that are more accessible. Over time, MedED has also established a community of “giving back”, with past student-attendees finding inspiration in their near-peers to become student-teachers themselves, establishing a sustainable chain of volunteers to maintain the Society running. To further enhance its value, considering student-staff partnerships may support student-teachers with their professional development and alleviate the burden of content creation, whilst also giving faculty an opportunity to use MedED as formal support resource for students and ensuring the quality of resources used are always of sufficient standard. Encouraging further student-student collaborations in developing resources and sharing feedback can also be of benefit to student-teachers by reducing workload, and ensuring the flexibility of session timings or locations could also make MedED more accessible for a diverse student body. Exploring the role that MedED could, and should, hold when it comes to supporting student wellbeing is also crucial. Overall, MedED stands as a testament to the value of student-led initiatives in medical education, offering a supportive, enriching, and inclusive environment that complements the formal curriculum and promotes both academic and personal growth.

## Supplementary Information

Supplementary Material 1.

Supplementary Material 2.

## Data Availability

The datasets for the study are available from the corresponding author upon reasonable request.

## Abbreviations

ICSM: Imperial College School of Medicine
MedED: Medical Education Society (referring to the student-led society at Imperial College School of Medicine)
OSCE: Objective Structured Clinical Examination (undertaken by Year 3/Phase 3a students at Imperial College School of Medicine, at the time of this work)
PACES: Practical Assessment of Clinical Examination Skills (undertaken by Year 5 and Year 6 students at Imperial College School of Medicine, at the time of this work)
MBBS: Bachelor of Medicine, Bachelor of Surgery
