# Supplementary material for: The value of a peer-to-peer teaching community in medical education
Source: BMC Med Educ. 2026 Jan 31;26:349. doi: 10.1186/s12909-026-08642-9 (PMC12934038; doi:10.1186/s12909-026-08642-9)
Supplement: Supplementary file 1 — Supplementary Material 1. [file 12909_2026_8642_MOESM1_ESM.docx]

**Supplement 1: Survey questions**

***Survey participant information sheet placed before the survey begins, on a separate page. Questions only visible after participant click “next” upon reading the information sheet.***

***1. Please state which of the following options describes you best:***

***Gender:***

- Female
- Male
- Non-binary
- Other
- Prefer not to say

***Year in medical school:***

- Phase 1a
- Phase 2b
- Phase 1c
- Year 4 (iBSc)
- Year 5
- Year 6

***2. Have you ever attended a MedED event, as a student?***

- Yes
- No

***If yes: redirected to question 3***

***If no: redirected to question 13***

***3. Which MedED lecture series and/or mentorship schemes have you attended this year?***

[Free text]

***4. How regularly did you attend MedED lectures this year?***

- Up to 25% of lectures
- Around half of lectures
- Over 75% of lectures

***5. How many mentorship scheme tutorials have you attended this year?***

[Free text]

***6. Please state to what extent you agree/disagree with the following statements*:***

Attending MedED events helped develop my medical knowledge.

Attending MedED events will shape me into a more competent doctor in the future.

Attending MedED event has given me helpful skills.

Attending MedED event has improved my confidence as a medical student.

Attending MedED event has improved my motivation as a medical student.

Attending MedED event has made me feel more academically supported as a medical student.

Attending MedED events made me feel part of a community of medical students.

**Each statement had the following options: strongly agree, somewhat agree, neither agree nor disagree, somewhat disagree, strongly disagree*

***7. What mode of lecturing do you prefer?***

- Online
- Either
- In-person

***8. What type of teaching do you prefer?***

- Large group
- Either
- Small group

***9. What motivated you to attend MedED events?***

[Free text]

***10. How do you think attending MedED events has benefitted you (including knowledge, self-confidence and wellbeing)?***

[Free text]

***11. How could MedED have supported you better (including knowledge, self-confidence and wellbeing)?***

[Free text]

***12. If you wish to add anything else about attending MedED events, please do so here.***

[Free text]

***13. Have you ever been a teacher for MedED? (including giving a lecture, being a mentor or lecture lead)***

- Yes
- No

***If yes: redirected to question 14***

***If no: end of survey***

***14. Please state which teaching roles you have had in MedED (please tick all that apply):***

- Lecture Series Lead
- Lecturer
- Mentor

***15. How many lectures or tutorials have you given at medical school?***

[Free text]

***16. What lecture series or mentorship schemes have you taught in?***

[Free text]

***17. Please state to what extent you agree/disagree with the following statements*:***

Teaching with MedED helped develop my medical knowledge

Teaching with MedED helped develop confidence in my medical knowledge.

Teaching with MedED helped develop confidence as a teacher.

Teaching with MedED will shape me into a more competent doctor in the future.

Teaching with MedED has enabled me to practice professionally transferable skills.

Teaching with MedED made me feel part of a community of medical students.

**Each statement had the following options: strongly agree, somewhat agree, neither agree nor disagree, somewhat disagree, strongly disagree*

***18. What mode of teaching do you prefer (as a teacher/mentor)?***

- Online
- Either
- In-person

***19. What type of teaching do you prefer (as a teacher/mentor)?***

- Large group
- Either
- Small group

***20. Please name key skills (if any) that teaching with MedED helped you develop.***

[Free text]

***21. What motivated you to become a MedED lecturer/mentor?***

[Free text]

***22. What were the challenges of being involved with MedED as a lecturer/mentor?***

[Free text]

***23. If you wish to add anything else about teaching/mentoring with MedED, please do so here.***

[Free text]
