# Supplementary material for: The value of a peer-to-peer teaching community in medical education
Source: BMC Med Educ. 2026 Jan 31;26:349. doi: 10.1186/s12909-026-08642-9 (PMC12934038; doi:10.1186/s12909-026-08642-9)
Supplement: Supplementary file 2 — Supplementary Material 2. [file 12909_2026_8642_MOESM2_ESM.docx]

**Supplement 2: Interview Guide**

***Interview Questions for Student-attendees***

1. What MedED events have you attended?

2. What made you attend these events?

3. What were the benefits of attending MedED events?

*Follow up: What differences did you notice between in-person and online lecturing?*

4. What stopped you from attending MedED events?

Follow up: What differences did you notice between in-person and online lecturing?

5. How has attending MedED events changed you as a medical student, if at all?

6. How do you think attending MedED events will impact your future career, if at all?

7. How else do you think MedED could help you?

***Interview Questions for Student-teachers***

1. What MedED events have you taught at?

2. What made you teach for these events?

3. What were the benefits of teaching in MedED events?

*Follow up: What differences did you notice between in-person and online teaching?*

4. What stopped you from teaching in other MedED events?

*Follow up: What differences did you notice between in-person and online teaching?*

5. How has teaching in MedED events changed you as a medical student, if at all?

6. How do you think teaching in MedED events will impact your future career, if at all?

7. How else do you think MedED could help you?
